# Proinflammatory and Oxidative Stress Markers in Patients with Periodontal Disease

**DOI:** 10.1155/2007/45794

**Published:** 2007-09-12

**Authors:** Ivan Borges Jr., Emília Addison Machado Moreira, Danilo Wilhem Filho, Tiago Bittencourt de Oliveira, Marcelo Barreto Spirelle da Silva, Tânia Silvia Fröde

**Affiliations:** ^1^Department of Nutrition, Federal University of Santa Catarina, 88040-970 Florianópolis, SC, Brazil; ^2^Department of Ecology and Zoology, Federal University of Santa Catarina,88040-970 Florianópolis, SC, Brazil; ^3^Department of Clinical Analysis, Federal University of Santa Catarina, 88040-970 Florianópolis, SC, Brazil

## Abstract

*Objective*. To evaluate the involvement of proinflammatory and oxidative stress markers in gingival tissue in individuals with chronic periodontitis. *Subject and methods*. Eighteen subjects were divided in two groups: experimental (age 52.9±5.0) and control (age 51.1±9.6). The activities of enzymatic antioxidants such as catalase, glutathione peroxidase (GPx), glutathione S-transferase (GST), glutathione reductase, nonenzymatic antioxidants: total glutathione and reduced glutathione, oxidized glutathione (GSSG), thiobarbituric acid reactive substances (TBARS), and myeloperoxidase activity (MPO) were evaluated in gingival tissues from interproximal sites. Statistical differences between groups were determined by independent Student t test and P<.05. *Results*. Individuals with periodontal disease exhibited a significant increase in the activities of MPO, GPx, GST, and also in TBARS and GSSG levels in gingival tissue compared to the control group (P<.05). *Conclusion*. The results of the present work showed an important correlation between oxidative stress biomarkers and periodontal disease.

## 1. INTRODUCTION

The inflammatory and immune reactions induced by the bacterial plaque represent the main characteristics of periodontitis, and this disease represents a particularly cogent example of problem arising from the phenomenon [1].

Studies have demonstrated that periodontal disease affects between 10% and 15% of the world's population, representing the greatest cause of tooth loss [2].

There is strong evidence that this disease affects a specific, predisposed group of the population that presents an exacerbated inflammatory/immune response to the periodontopathogenic bacteria that accumulate on the teeth and around the gingival tissue, which in turn may lead to tissue damage [1, 3].

The exact mechanism of periodontitis development, including the prior agents or mediators involved, is not clear. Periodontitis manifests itself as a multifactor phenomenon, including the generation of reactive oxygen species (ROS) [4].

The strong evidence linking ROS to the pathological destruction of the connective tissue during periodontal disease rests on the presence of neutrophils infiltration as the main event in the host's response to bacterial invasion [1, 5, 6]. Furthermore, hydroxyl radical (^*•*^OH) is most active in damaging important molecules such as DNA proteins and lipids, while hydrogen peroxide (H_2_O_2_), even not being considered a potent ROS, is capable of crossing the nuclear membrane and also damaging the DNA [7]. Quantitatively, the main source of superoxide anion (${\text{O}}_{2}{}^{•-}$) and other ROS responsible for initiation reactions is the respiratory chain. However, its presence in the periodontal tissue results first and foremost from the activation of phagocytes (neutrophils and macrophages), such as antibacterial agents [1, 5, 8]. It has been suggested that superoxide anion is involved in bone reabsorption which has been corroborated by studies that have demonstrated the presence of this anion in reabsorption zones adjacent to the osteoclasts [9].

The hydroxyl radical is able to initiate a classical chain reaction known as lipid peroxidation leading to the vasodilation production and rat bone reabsorption [8]. An example of the damage caused by hydrogen peroxide is that it can stimulate the phosphorylation of the NF${\text{-}}_{K}$B-${\text{I}}_{K}$B complex activating the NK${\text{-}}_{K}$B and facilitating nuclear translocation and downstream of proinflammatory cytokines, including interleukin-2 (IL-2), interleukin-6 (IL-6), interleukin-8 (IL-8), β-interferon, and tumor necrosis factor-α (TNF-α) that are very important in the pathogenesis of periodontal disease [10–12].

ROS production is inevitable in all aerobic organisms including humans, who necessarily posses a complex system of antioxidant defense [8, 13]. If the homeostasis is interrupted in favor of ROS, an oxidative stress situation is created [13].

The aim of this study was to evaluate the involvement of proinflammatory and oxidative stress markers in individuals with chronic periodontal disease.

## 2. SUBJECTS AND METHODS

### 2.1. Experimental design

This case-control study was carried out between May–September, 2003 in a single centre (Peridontologic Centre, Florianopolis, Santa Catarina, Brazil). Eighteen subjects were divided into 2 groups: experimental group (E), comprising individuals with chronic periodontitis, age 52.9±5.0 (4 men and 5 women), with the following inclusion criteria: presence of chronic inflammation (pain, redness, heat, swelling), diagnosed according to bleeding on probing, at least 5 or 6 sites with probing depth ≥5 mm, attachment loss ≥3 mm, and extensive radiographic bone loss [14]. Control group (C), was composed of healthy individuals, age 51.1±9.6 (4 men and 5 women) with no prior history of periodontal disease. The probing depth in this latter group did not exceed 3 mm [14].

For both groups, the following items were considered as exclusion criteria: infection, cardiovascular and/or neurological illness, renal insufficiency and/or diabetes; pregnancy; smoking; use of antibiotics and/or hormonal or nonhormonal anti-inflammatory drugs 6 months prior to tissue collection.

In the day of the surgical procedures, the patients showed no complication.

This study was in agreement with the ethical principles of the World Medical Association Declaration of Helsinki (1964). Permission for this study was obtained from the Ethical Committee for Human Research of the Federal University of Santa Catarina (Project no. 210/2002) and the study included only individuals that agreed to participate after reading and signing a free and informed consent form, except those with difficulty in understanding and communicating, with physical handicap, or both, which could have compromised the sample collection.

### 2.2. Assessment of periodontal disease

All of the surgical procedures were assessed and performed by a periodontist, according to the necessity for each treatment. The patients with chronic periodontitis (experimental group) were submitted a pocket depth reduction technique from palatal/lingual, buccal, and interproximal sites [15]. The biopsies were obtained from inflammatory granulation tissues, connective and epithelium tissues.

The samples collected from the control group were obtained from quarantined mucosa during the surgical procedures of impacted third molars removed following orthodontic recommendation or after the reopening of dental implants.

All samples from the experimental and control groups were removed during the surgical and were immediately frozen in liquid nitrogen ($≅-170^{{}^{\circ}}$C) subsequently latter laboratory analysis.

The determinations of the inflammatory parameters were made as scheduled on different days. On the day of the experiments, the samples were deathward at room temperature to determine the different parameters: CAT, GPx, GST, GR, MPO activities, and the contents of TG, GSH, GSSG, and TBARS.

### 2.3. Assessment of antioxidants enzymes

The method described by Aebi was employed to measure the catalase activity (CAT) by measuring the decay of a freshly prepared 10 mM hydrogen peroxide solution at 240 nm [16].

Glutathione peroxidase (GPx) was measured at 340 nm through the glutathione/NADPH/glutathione reductase system by the dismutation of *tert*-butyl hydroperoxide [17].

Glutathione S-transferase (GST) activity was determined at 340 nm using CDNB (1-chloro-2, 4-dinitrobenzene) as substrate and a 0.15 M GSH concentration [18].

Finally, glutathione reductase (GR) activity was assayed at 340 nm by measuring the rate of NADPH oxidation [19]. Results were expressed as mmol^−1^g^−1^(CAT) and μmol min^−1^g^−1^ (GPx, GST, and GR).

### 2.4. Nonenzymatic antioxidants

Nonprotein thiols, mostly present as the reduced form of glutathione (GSH), were measured at 412 nm according to Beutler using Elmann's reagent (DTNB: 2-dithionitrobenzoic acid) [20]. Immediately after thawing, acid extracts were obtained by adding tissue portions to 12% trichloroacetic acid (1 : 4 w/v), which were then centrifuged at 15 000 g for 5 minutes at $5^{{}^{\circ}}$C. Supernatants from the acid extracts were added to a buffer containing 0.25 mM DTNB in 0.1 M Na_2_PO_4_, pH 8.0, and the formation of the thiolate anion was immediately determined. Total glutathione (TG) was also measured at 412 nm in acid extracts according to the enzymatic method of Tietze [21]. Oxidized glutathione (GSSG) was also determined by calculating the difference (in equivalents of GSH) between total glutathione and reduced glutathione contents. Results were expressed as μmol g^−1^.

### 2.5. Myeloperoxidase activity

Myeloperoxidase activity was measured according to the method developed by Rao et al., and was estimated by colorimetric measurement at 450 nm on an Elisa plate reader [22]. Results were expressed as mU/mL.

### 2.6. Lipoperoxidation assay

Thiobarbituric acid-reactive substance (TBARS) contents were determined to assess endogenous lipid oxidation in gingival tissue according to Ohkawa et al. [23] and Bird and Draper [24]. After thawing, gingival portions were immediately added to 12% trichloroacetic acid (1 : 4 v/v) and were then centrifuged at 15 000 g for 5 minutes at 5°C. Supernatants were added to 50 mM Tris-HCl pH 7.0, vortexed for 20 seconds, added to 0.67% (w/v) 2-thiobarbituric acid, maintained in boiling water for 60 minutes, cooled at $5^{{}^{\circ}}$C for 30 minutes, and then analyzed spectrophotometrically at 535 nm. Concentrations were expressed as nmol TBARS/g wet tissue using $\in_{535}=153$ mM^−1^cm^−1^.

All the biochemical parameters described above were measured in duplicate, except for the TBARS determinations, which were measured in triplicate.

## 3. STATISTICAL ANALYSIS

The results were expressed as mean ± SEM. Statistical differences between groups were determined by independent Student t test analysis. For all analyses, P<.05 was used to assess overall differences.

## 4. RESULTS

### 4.1. Antioxidant enzyme activities

These results show that there were no significant differences in catalase activity in the experimental group (E) compared to the control group (P=.523). However, a significant increase in GPx activities in the experimental group when compare to the control group (P=.006) was detected (see Table 1). Furthermore, in the experimental group, a significant increase of glutathione S-transferase (GST) values compared to the control group (P=.001) was also observed (see Table 1). The analysis of glutathione reductase (GR) revealed no differences between the studied groups (P=.481) (see Table 1).

### 4.2. Myeloperoxidase activity

The myeloperoxidase activity revealed a significant increase of this inflammatory biomarker in the experimental group (P=.003) (see Table 1).

### 4.3. Nonenzymatic antioxidant defenses

In relation to the total glutathione (TG) and the reduced glutathione (GSH) contents, no differences in the values of the experimental group compared to the control group were found (P>.05) (Table 1). However, the values obtained for oxidized glutathione (GSSG) showed a significant increase in the experimental group when compared to the control group (P=.019) (see Table 1).

### 4.4. Measurement of tissue lipoperoxidation

Lipoperoxidation was measured through TBARS contents, which were significantly a higher increase in the experimental group (P=.015) (see Table 1).

## 5. DISCUSSION

Few studies have considered the effect of the imbalance between oxidants and antioxidants in patients with periodontitis, which in turn predisposes such individuals to the damaging effects of ROS in the periodontium [25]. Ellis and collaborators analyzed gingival tissues from patients with severe periodontal disease and showed that the activity of catalase was decreased [26]. In the present study, the activity of catalase was not different when the experimental and control groups were compared. One possible explanation for these different responses is that the patients with periodontal disease were in distinct stages of the disease. In this regard, it is well known that the antioxidant responses found in different pathologies depend on the severity or extension suffered by the patients, and long-term chronic conditions may have jeopardized the antioxidant defenses [8].

The analysis of the enzyme glutathione peroxidase revealed a significant increase in the experimental group. A GPx increase in gingival samples from dogs and humans with periodontal disease has already been described [6, 27]. The GPx increase may represent possible antioxidant compensation in detoxification reactions of organic peroxides produced during oxidative stress in gingival tissue [28]. 

Furthermore, glutathione S-transferase (GST) also revealed a significant increase in its activities in the experimental group. Since GST has a direct role in the neutralization of hydroperoxides derived from the lipoperoxidation processes, increases in GST activities are probably related to the oxidative stress caused by the periodontal inflammatory process [8, 29]. GST comprises a group of enzymes that are also able to detoxify a variety of compounds including xenobiotics derived from pathogenic microorganisms, catalyzing their conjugation with GSH [30]. Hence, increases in GST activities are excellent indicators of endogenous detoxification from exogenous sources [31]. 

The enzyme glutathione reductase (GR) has an important accessory antioxidant function related to glutathione peroxidase and glutathione S-transferase. GR intervention continuously regenerates GSH from GSSG in the presence of NADPH, therefore preventing cellular loss of GSH [32]. However, in the current study, no differences in GR activities were detected in gingival tissue between the two groups. 

The ubiquitous tripeptide glutathione (GSH) acts directly as a generic ROS scavenger or as a cofactor of GPx and GST, either by catalyzing the reduction of hydrogen peroxide and lipid hydroperoxides or by the conjugation/excretion processes of the so-called Phase II reactions [31]. Total and reduced glutathione revealed a tendency to increase, but the values were not significantly different in patients with periodontitis compared to the controls. 

Despite GSH, these results suggest a de novo synthesis of glutathione, which is extremely necessary for the homeostasis of cells [31]. Some periodontopathogenic bacteria deplete GSH, and this may explain the amount of this antioxidant was not elevated in the gingival tissue of patients with periodontitis combined with an increase of the GPx activity in the affected tissue [33, 34]. A similar result was obtained in gingival tissue and blood, but lower levels of GSH were detected in the crevicular gingival fluid of patients with chronic periodontitis, when compared to normal subjects [27, 35]. 

On the other hand, a significant increase in GSSG concentrations was detected in the experimental group, which is a clear biomarker of oxidative stress detected in inflammatory processes linked to periodontitis. Nevertheless, Chapple et al. (2002) found less GSSG in gingival cervical fluid of patients with chronic periodontitis [35]. Consistent with the results for GSSG, tissue lipoperoxidation, measured as TBARS contents in the gingival tissue, also displayed a significant increase (P=.015) in individuals affected by periodontitis, and oxidative stress, in the gingival tissue associated with periodontal disease [36]. 

The systemic depletion of antioxidants clearly indicates that in chronic periodontitis the antioxidant system is affected by a relatively strong oxidation insult, which can also deplete nutritional antioxidants such as vitamin E and C in plasma and also vitamin E in red cell membrane [27].

Moreover, myeloperoxidase activity in gingival tissue showed a significant increase in patients with periodontal disease when compared to the control group, an indicative of a chronic inflammatory process also reflected at a systemic level. These results were similar to the measurements obtained from the analysis of crevicular gingival fluid in humans with periodontal disease [37].

Oxidative stress processes and alterations in the immune system are closely related and have been described in different diseases, thus both the aspects also seem to be linked to the pathogenesis of periodontal disease, and can also be detected in the plasma of patients with periodontitis [8, 27, 35]. However, the extent to which ROS overgeneration influences the initiation and progression of periodontal diseases is still unknown.

In conclusion, in spite of the limited number of samples examined in the present study, the results indicate a relationship between proinflammatory and oxidative stress biomarkers and periodontal disease.

## Figures and Tables

**Table 1:** Biomarkers of oxidative stress in gingival tissue of healthy control and individuals with periodontal disease. The results are represented by means ± SEM of controls (healthy individuals) and patients with periodontal diseases (E). CAT = Activities of Catalase, GPx = glutathione peroxidase, GST = glutathione S-transferase, GR = glutathione reductase, MPO = myeloperoxidase, TG = total glutathione, GSH = glutathione.

	Control	Experimental	P
CAT (mmol min^−1^g^−1^)	1.64±0.46	2.00±0.30	.523
GPx (μmol min^−1^g^−1^)	0.80±0.11	2.09±0.34	.006***
GST (μmol min^−1^g^−1^)	4.28±0.89	10.81±0.63	.001***
GR (μmol min^−1^g^−1^)	0.28±0.04	0.22±0.06	.481
MPO (mU/mL)	222.20±54.00	556.44±76.77	.003***
TG (μmol g^−1^)	0.44±0.11	0.63±0.09	.171
GSH (μmol g^−1^)	0.38±0.08	0.46±0.06	.459
SSG (μmol g^−1^)	0.06±0.01	0.17±0.04	.019***
TBARS (nmol g^−1^)	113.07±16.59	188.80±20.73	.015***

***(P<.001) mean statistical differences between controls and experimental group.
